# An Integrated Gold-Film Temperature Sensor for In Situ Temperature Measurement of a High-Precision MEMS Accelerometer

**DOI:** 10.3390/s20133652

**Published:** 2020-06-29

**Authors:** Xiaoxiao Song, Huafeng Liu, Yanyan Fang, Chun Zhao, Ziqiang Qu, Qiu Wang, Liang-Cheng Tu

**Affiliations:** 1 MOE Key Laboratory of Fundamental Physical Quantities Measurement & Hubei Key Laboratory of Gravitation and Quantum Physics, PGMF and School of Physics, Huazhong University of Science and Technology, Wuhan 430074, China; songxx@hust.edu.cn (X.S.); fangyanyan@hust.edu.cn (Y.F.); chun_zhao@hust.edu.cn (C.Z.); ziqiangqu@hust.edu.cn (Z.Q.); wangqiu@hust.edu.cn (Q.W.); tlc@hust.edu.cn (L.-C.T.); 2 TianQin Research Center for Gravitational Physics and School of Physics and Astronomy, Sun Yat-sen University (Zhuhai Campus), Zhuhai 519082, China

**Keywords:** temperature sensor, in situ temperature measurement, integrated, high-precision MEMS accelerometer

## Abstract

Temperature sensors are one of the most important types of sensors, and are employed in many applications, including consumer electronics, automobiles and environmental monitoring. Due to the need to simultaneously measure temperature and other physical quantities, it is often desirable to integrate temperature sensors with other physical sensors, including accelerometers. In this study, we introduce an integrated gold-film resistor-type temperature sensor for in situ temperature measurement of a high-precision MEMS accelerometer. Gold was chosen as the material of the temperature sensor, for both its great resistance to oxidation and its better compatibility with our in-house capacitive accelerometer micro-fabrication process. The proposed temperature sensor was first calibrated and then evaluated. Experimental results showed the temperature measurement accuracy to be 0.08 °C; the discrepancies among the sensors were within 0.02 °C; the repeatability within seven days was 0.03 °C; the noise floor was 1 mK/√Hz@0.01 Hz and 100 μK/√Hz@0.5 Hz. The integration test with a MEMS accelerometer showed that by subtracting the temperature effect, the bias stability within 46 h for the accelerometer could be improved from 2.15 μg to 640 ng. This demonstrates the capability of measuring temperature in situ with the potential to eliminate the temperature effects of the MEMS accelerometer through system-level compensation.

## 1. Introduction

The advancement and innovation of modern science and technology benefit on the development of sensing technology. Sensors are widely used in many fields such as consumer electronics, automobiles, aerospace, manufacturing and environmental monitoring [1]. Temperature sensors are one of the most important types of sensors used in those fields with the requirement of precise temperature monitoring. According to sensing methods, temperature sensors can be categorized into contact temperature sensors and non-contact temperature sensors. The non-contact temperature sensor is mainly a thermal radiation thermometer. According to the sensing mechanisms, the existing contact temperature sensors can sub-categorized into thermistor, thermocouple, platinum resistor and micro temperature sensors. Thermistor temperature sensors are usually droplet, cylindrical or thin-film metal oxide sensors. The internal resistance decreases or increases as the temperature rises; therefore, they can be divided into negative temperature coefficient (NTC) thermistors and positive temperature coefficient (PTC) thermistors, respectively. However, the linearity of thermistors is poor, and the accuracy is poor. Thermocouple sensors are the most widely used temperature sensors in industrial measurement. They directly contact the measured objects; therefore, are not affected by the intermediate medium and have high accuracy. Platinum resistors are most widely used in laboratory and have higher accuracy than the former two counterparts, but they are expensive, and the application is limited.

In many fields, it is often necessary to simultaneously measure parameters such as acceleration, pressure, temperature and magnetic field. However, in these applications, due to the strict limitations of environmental adaptability, room restriction and costs, the sensor is required to be miniaturized. The complementary metal oxide semiconductor (CMOS) technology based micro temperature sensors are widely used in industrial and automotive fields due to small-size, high-performance and low-cost. In the last few decades, the development of microelectromechanical systems (MEMS) technology has driven the development of sensors towards miniaturization, high integration and diversification [2,3,4,5,6,7,8]. There are many types of micro temperature sensors currently developed using MEMS technology, including capacitive, piezo-resistive, tunneling, resonant and thin-film thermal-sensitive resistance temperature sensors. A MEMS capacitive temperature sensor [9] consists of a bimetallic micro-cantilever and a comb drive structure. When the temperature changes, the bimetallic micro-beam is deformed due to thermal mismatching and the effective capacitance area of the comb capacitor changes; therefore, temperature can be measured by the capacitive readout circuit. A MEMS piezo-resistive temperature sensor with silicon micro-bridge structures [10] has a thicker silicon film and a lower sensitivity. However, the sensitivity can be improved by reducing the thickness of silicon films, it will increase the difficulty of production and increase costs. The MEMS tunneling thermometer based on tip deflection of the bimetallic cantilever beam [11] has a high sensitivity, but its temperature measurement range is very small, and it is difficult to conduct batch processing.

One of the development trends of the MEMS-based sensors is to integrate more sensors on a single chip to detect different physical quantities at the same time and has small volume and low unit cost. To date, many studies have been made on the integration of multiple sensors using MEMS technology. The SiTime team has published a MEMS resonator-based clock with the accuracy of 0.1 ppb. It has an integrated micro temperature sensor that performs temperature measurements by two independent resonators with different temperature coefficients, and the difference of resonant frequencies varies with temperature fluctuation. This resonant temperature sensor has a resolution of 20 μK, which is the state-of-the-art in the world [12,13]. A vibrating-beam accelerometer with a resolution of 50 ng combined with an integrated resonating temperature sensor has been investigated [14]. The thermal transient behavior of the temperature sensor has been studied using finite element analysis (FEA) without actual test result. However, resonant temperature sensors also have disadvantages, such as complex conditioning circuits and the possibility of vibration crosstalk with the measured object. The thin-film thermal resistance temperature sensors have relatively simple deposition process therefore have been widely used. They have the advantages of a good long-term stability and a good repeatability, rapid thermal response, high temperature coefficient of resistance (TCR), excellent linearity, a large measurement range and a reasonably high resolution [15]. For example, a Pt thin-film temperature sensor annealed at 800 °C for two hours has a TCR of 2.40 × 10^−3^/°C [16]. Moreover, the thin-film thermal resistance temperature sensors have the potential to be integrated on MEMS mechanical sensors with compatible processes.

In this study, an integrated on-chip temperature sensor, which is compatible with MEMS processes, was introduced for in situ and high-resolution temperature measurement of the main MEMS sensor. It aims to solve the issues of temperature gradient, thermal transmission time and low-measurement resolution, which are the main errors of traditional temperature compensation for high-precision MEMS sensors. The gold-film temperature sensors with different dimensions are designed and fabricated. The gold-film temperature sensor with the conditioning circuit was first calibrated. Then, several experiments were performed to investigate the performance of the gold-film temperature sensors, including consistency, repeatability and the intrinsic noise floor. The proposed gold-film temperature sensor was integrated on a high-precision MEMS accelerometer for in situ temperature measurement.

## 2. Design and Fabrication

The working principle of the proposed gold-film temperature sensor was that the resistance exhibits to the flow of an electric current was related to its temperature. Depending upon the accuracy to after, the relationship governing resistance temperature sensor output against temperature follows the quadratic equation:

(1) $$R_{T}/R_{0}=1+\alpha T+\beta T^{2},$$

where *R_T_* and *R_0_* are, respectively the resistance of the resistor at *T* °C and 0 °C, α is the first-order temperature coefficient of resistance (TCR) and *β* is the second-order coefficient. For a small temperature measurement range, the first-order approximation can be adequate.

### 2.1. Material Selection

The design rational of the thin-film temperature sensors was to be capable of being integrated with MEMS sensors. Therefore, the materials and fabrication processes needed be compatible with MEMS sensors. Platinum resistance temperature detector (RTD)—such as Pt100—is the most widely used in laboratory for accuracy temperature measurement. However, several materials could fulfil the basic requirement of providing a predictable, smooth and stable temperature with resistance relationship, such as copper, gold, nickel and silver, the selection of platinum lies in the inherently high electrical resistivity values. However, for the MEMS fabrication, the resistance of the thin film resistor could be increased by a thinner film or a longer trace. Compared with copper, nickel and silver, gold as a noble metal had a much greater resistance to oxidation. Compared with platinum, gold was more commonly used in MEMS fabrication and compatible to most of the processes. Therefore, gold was more suitable for integrated temperature sensors on MEMS sensors.

### 2.2. Shape Design

Apart from the temperature fluctuation, the strain due to differential expansion between the thin-film resistor and its surroundings could also affect the resistance, which was a challenge for the thin-film temperature sensor design. The design rational was to obtain a sufficient resistance value with a minimum strain. The resistance of the gold thin-film resistor can be expressed by the following basic formula:

(2) R=ρL/A,

where ρ is the resistivity of gold, *L* is the length of gold trace, *A* is the cross sectional area of the gold trace. In order to meet the design rational, the thin-film temperature sensor should be deployed in a minimum area for a small strain. The resistance should be maximized by increasing the actual length meanwhile decreasing the cross-sectional area of the gold trace. The proposed shape is a planar coil with multiple parallel traces. The length of a single trace is *L*; the number of traces is *N*, the total width of the coil winding is *s*, so the total length of the coil can be expressed as:

(3) L=N×l+s.

According to the literature [17], once the thickness of the thin-film metal resistor less than 1 µm, the thermal response lag can be ignored. Considering the cost of gold deposition and lift-off fabrication tolerance, the thickness of the gold trace *t* is chosen to be 0.1 μm, and the width of the gold trace *w* is chosen to be 10 μm. The design parameters of different prototype chips are shown in Table 1. Chips A, B, C, D and E have the same trace thickness and width and the same single trace length of about 3.3 mm and coil winding width of about 3.7 mm but have different trace numbers of 10 to 50. Therefore, the total length of the coil and the nominal resistance can be calculated by Equations (3) and (2), respectively, which are also shown in Table 1. The resistivity of the gold thin-film resistor ρ at room temperature was 2.40 × 10^−8^ Ω⋅m, so that the resistance of the Chip A could be derived as 871 Ω. With the known standard TCR of gold block of 3.2 × 10^−3^/°C, when temperature changed by 0.001 °C, the resistance of the gold-film resistor changed by about 0.003 Ω. However, when temperature changes, *A*, *L* and ρ all changes. Since the changes of *A* and *L* were very small compared with the change of ρ, so it was always assumed that only the resistivity of metal materials ρ changes.

### 2.3. Fabrication Process

Since the resistance of an electrical conductor will experience variations by electron scattering effects and atomic lattice vibrations due to the impurities and lattice defects, an important requirement for accurate resistance thermometry was that the sensing element needed to be pure. The challenge for fabrication was to support the fine, pure thin-film adequately. Meanwhile, the thin-film needed also be (and remain) in an annealed condition, via suitable heat treatment of the materials such that it was not inclined to change physically. In addition, the thin-film needed to be kept in an environment protected from contamination so that chemical changes were indeed obviated.

**Figure 1:**
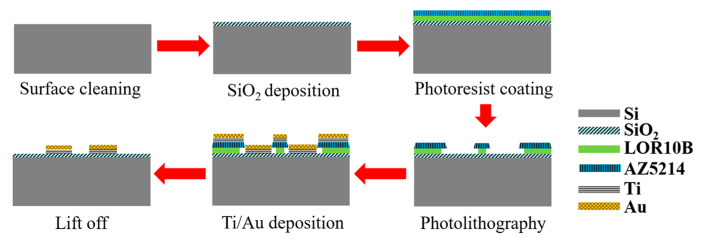
Schematic diagram of gold-film temperature sensor processing flow.

The proposed gold-film temperature sensor was fabricated based on E-beam evaporation with the gold target of at least 99.999% purity and the lift-off feature definition process. Prototypes were fabricated based on the substrate of 4-inch single-crystal silicon wafers with natural surface oxidation. The fabrication processing flow of the proposed gold-film temperature sensor is illustrated in Figure 1. The details of the processes are as follows: (1) the surface of the silicon wafer was cleaned by organic solutions and plasma cleaning to remove impurities and organic substances; (2) silicon dioxide was deposited on the surface of the silicon wafer by plasma enhanced chemical vapor deposition (PECVD) as an insulating layer between the metal thin-film and the silicon substrate; (3) the disc was uniformly spin-coated with double-layer photoresist (LOR10B & AZ5214); (4) a ATD1000 laser direct writer was used to define the patterns and transferring the patterns by AZ400 K developer; (5) an E-beam evaporation coater was used to deposit a titanium layer with a thickness of 20 nm and a gold layer with a thickness of 100 nm on the substrate surface, where the titanium layer was an adhesion layer between gold and silicon dioxide; (6) the sample wafer was immersed in acetone and then MIF solution to remove AZ5214 and LOR10B, respectively. In this case, the metal features on photoresist could be lifted off, leaving the required metal features on the substrate. In the end, the samples were be annealed at 300 °C for at least 1 h.

**Figure 2:**
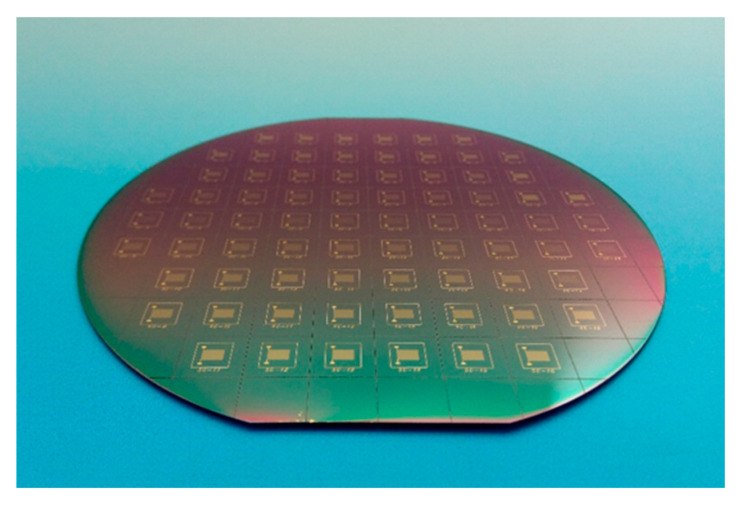
Gold-film temperature sensors fabricated on a 4-inch silicon wafer.

The fabricated wafer with gold-film temperature sensors is shown in Figure 2. The measured resistance values of the temperature sensors after processing are shown in Table 1, where each value was averaged from 8 chips of each type. Since the conductivity of titanium was only about 5% that of gold, the resistance reduction due to the titanium layer was ignored. In addition, the resistance of the wire-bonding pads for electrical connections was also ignored. The deviation between the calculated resistance and the measured resistance was about 10% which was reasonable due to the density difference between the deposited thin-film and the body block. There was no obvious resistance change before and after the annealing process. The gold-film temperature sensor chips were singulated by laser cutting. Each individual temperature sensor was connected to the adapter board by wire-bonding, and then connected to the temperature readout circuit board.

## 3. Experiments and Results

In order to comprehensively study the proposed gold-film temperature sensor, the corresponding readout circuit and the experiment setup are described, followed by sensor calibration and several performance evaluation experiments including consistency study, repeatability study and measurement resolution evaluation. Then, the application of the proposed temperature sensor for in situ temperature measurement of a high-precision MEMS accelerometer was demonstrated.

### 3.1. Experiment Setup

**Figure 3:**
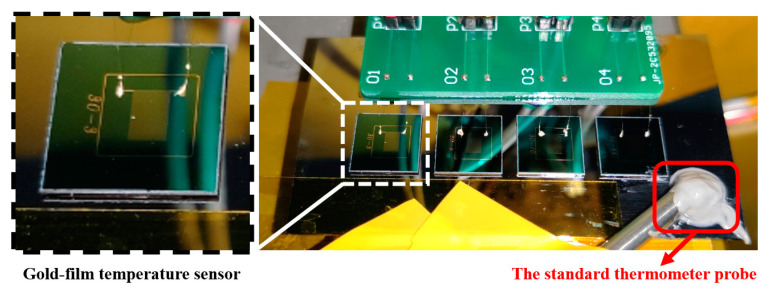
Experimental setup diagram inside the thermostat chamber.

The evaluation experiments of the fabricated gold-film temperature sensors were set up in a thermostat. In order to monitor the change of ambient temperature, a commercial standard thermometer (Model 1504 from FLUKE) was used. The gold-film temperature sensors and the standard thermometer for metrology were placed into the thermostat. The temperature readout circuit was placed outside the thermostat and was covered by a metal case for electromagnetic shielding. The temperature sensor chips and the standard thermometer probe (Model 5643-D from FLUKE) inside the thermostat chamber were shown in Figure 3. In order to ensure good heat conduction, all the temperature sensor chips and the thermometer probe were glued on the same silicon substrate. Depending on resistance and sensitivity, different chips were used for the following experiments, and the list was shown in Table 2. In order to suppress the electromagnetic interferences, a multiplayer shielding case was used in all experiments below.

### 3.2. Sensor Readout Circuit and Calibration

**Figure 4:**
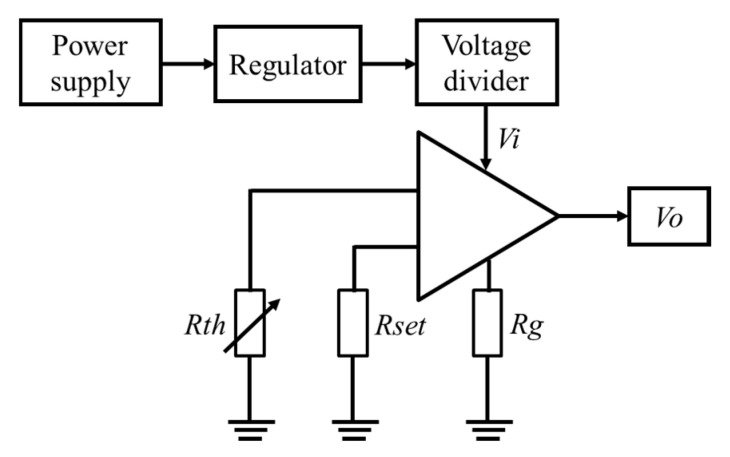
Schematic diagram of the temperature readout circuit.

The schematic diagram of the temperature readout circuit is illustrated in Figure 4. The circuit consisted of a voltage regulator, a voltage divider, a commercial thermistor signal amplifier (INA330) and precise resistors *R_set_* and *R_g_*. *R_th_* was the proposed temperature sensor. *R_set_* was the reference resistor whose value determines the middle point of the temperature measurement range. *R_g_* set the gain of the readout circuit. By changing the resistance of *R_set_* and *R_g_*, the temperature measurement range of the circuit could be adjusted. In the following experiments, all the sensors were configured with a temperature measurement range of 15 °C to 40 °C. A larger temperature measurement range, such as from −40 °C to 85 °C, could be obtained, however, with the cost of a lower temperature measurement sensitivity. Therefore, the appropriate *R_set_* and *R_g_* resistance was chosen according to the application-orientated temperature range.

According to the output formula of the temperature readout circuit:

(4) $$V_{o}=V_{i}\times \left(\frac{1}{R_{th}}-\frac{1}{R_{set}}\right)\times R_{g}+2.5,$$

The resistance of the temperature sensor can be derived:

(5) $$R_{th}=V_{i}\times R_{g}\times R_{set}/\left[R_{set}\times \left(V_{o}-2.5\right)+V_{i}\times R_{g}\right].$$

**Figure 5:**
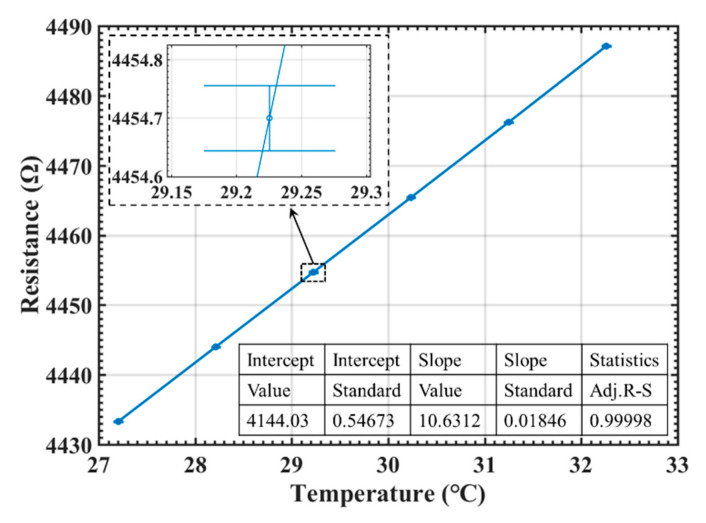
Fitting *R* and *T* curves.

The proposed temperature sensor chips were first calibrated. According to the required temperature range of 15 °C to 40 °C in laboratory, the thermostat was first set at the middle point of about 27.5 °C. Once the chamber temperature was stable, the temperature was increased to 32.5 °C with each step of 1 °C for 1 h. For each step, the later 30 min’ data, which was believed to be stabilized, was averaged and then fitted to the temperature readings from the standard thermometer. The fitting curve of resistance to temperature for Chip E-1 is illustrated in Figure 5. The calibrated first-order relationship between gold-film resistance *R_th_* and temperature *T* is

(6) $$R_{th}=4144.03\left(\pm 0.55\right)+10.63\left(\pm 0.02\right)\times T.$$

**Figure 6:**
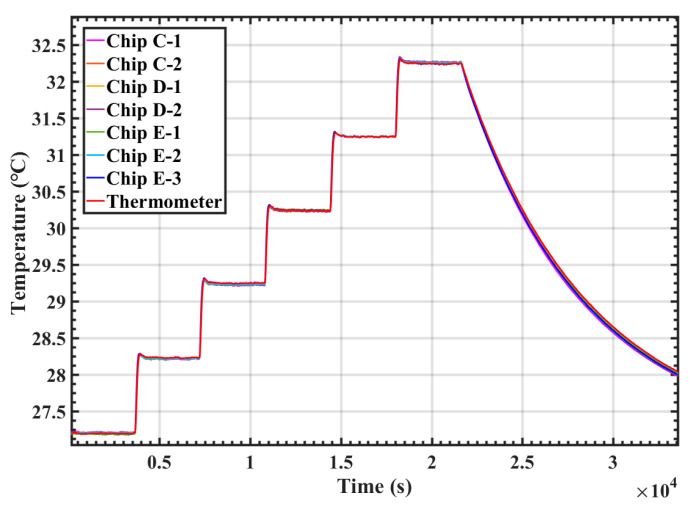
Comparison of temperature outputs of the proposed temperature sensors after calibration with the standard thermometer.

The TCR of the temperature sensors was 2.59 ± 0.05 × 10^−3^/°C that was calibrated from ten different sensors from Chip C, Chip D and Chip E. After converted by the calibrated TCR, the temperature measurement accuracy of the sensors can be evaluated to be 0.08 °C (where the accuracy of the standard thermometer is 0.0015 °C), as shown in Figure 6.

### 3.3. Consistency Study

**Figure 7:**
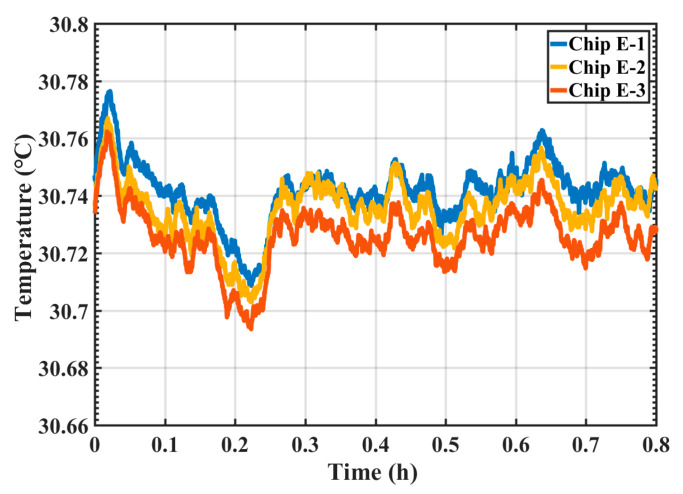
Consistency test results between Chip E-1, 2, 3.

Consistency represents the quality of the fabricated temperature sensors and evaluates how closely they agree with each other. In the consistency study experiment, three temperature sensors with a higher sensitivity, Chip E-1, Chip E-2 and Chip E-3 from the same batch were studied. The thermostat was set to 30 °C, and the sampling rate of the temperature readout circuit outputs was 1 Hz. The consistency test results are illustrated in Figure 7, showing that the difference between the temperature sensor chips was within 0.02 °C. The correlation coefficient of the temperature outputs between the fabricated temperature sensors and the standard thermometer was 0.95, which indicates the fabrication tolerance was reasonably good.

### 3.4. Repeatability Study

**Figure 8:**
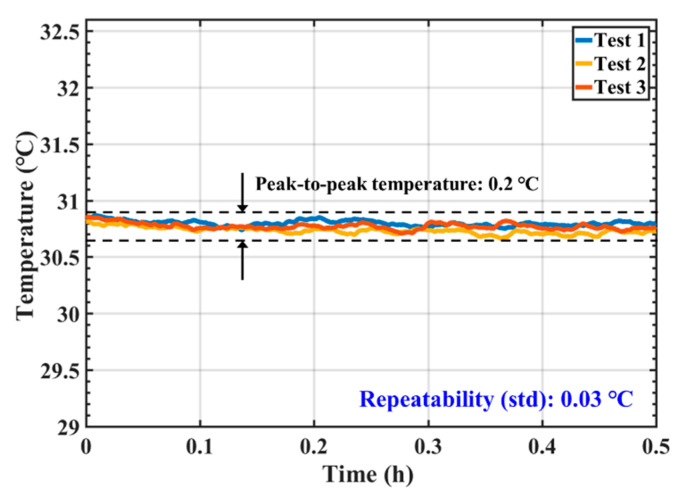
Repeatability study results for the same temperature sensor Chip E-1.

With the same experiment setup as the consistency experiment, the repeatability of the Chip E sensors was also investigated. Three tests were conducted: Test 1 and Test 2 were performed in the morning and afternoon of the same day. Test 3 was conducted 7 days after Test 1 and Test 2. The resulted of the repeatability experiment of Chip E-1 were shown in Figure 8. The average temperature of Test 1 was 30.798 °C with a standard deviation of 0.002 °C; the average temperature of Test 2 was 30.733 °C with a standard deviation of 0.003 °C; the average temperature of Test 3 was 30.772 °C with a standard deviation of 0.003 °C. The peak-to-peak temperature difference of the three measurements was 0.2 °C. The repeatability of Chip E-1 equals to the standard deviation of 0.03 °C. Other two Chip E sensors had the same performance. The good repeatability performance suggests the proposed temperature sensors were generally reliable.

### 3.5. Measurement Resolution

**Figure 9:**
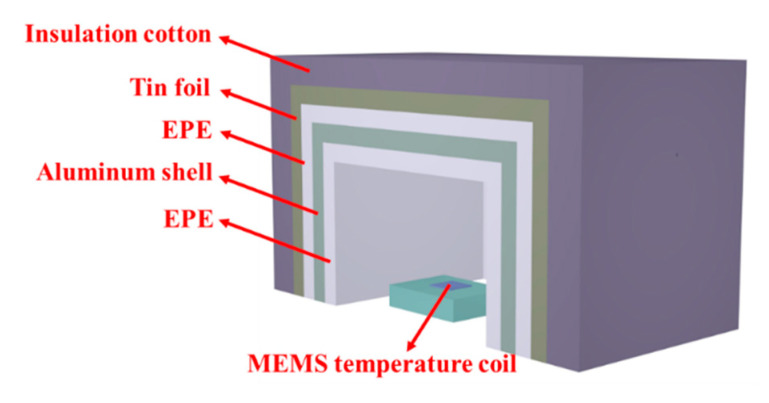
Schematic diagram of long-term noise floor test.

In order to evaluate temperature measurement resolution of the proposed gold-film temperature sensor, a noise floor evaluation experiment was conducted with the experiment setup shown in Figure 9. The Chip E-1 sensor was placed in a thermal insulation box with multiple layers, including the first expandable polyethylene (EPE) layer, an aluminum shell, the second EPE layer, a tin foil and insulation cotton from inside to outside. The thermal insulation box performs as a low-pass filter for cutting off random temperature fluctuations, such as doors open and close, people in and out. The temperature readout circuit was placed outside and shielded by a metal box. The experiment was conducted for about 6 days with a sampling rate of 1 Hz.

**Figure 10:**
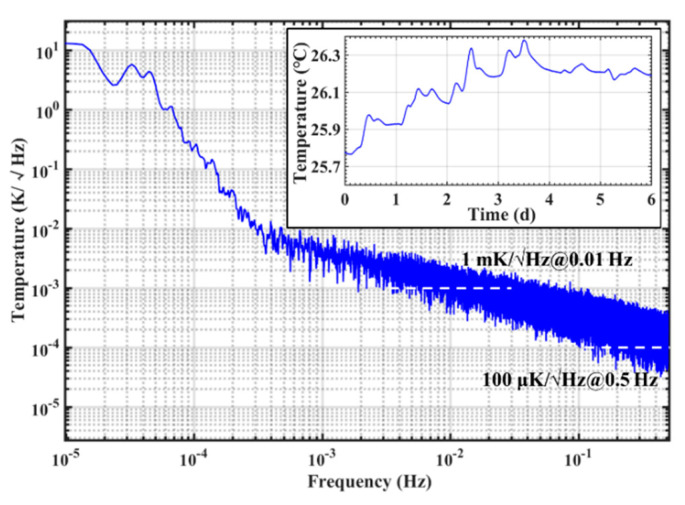
Noise floor test results of the gold-film temperature sensor Chip E-1.

The experimental results are shown in Figure 10. It can be seen that the entire temperature change was about 0.6 °C during 6 days. The noise floor of the proposed gold-film temperature sensor Chip E-1 was 1 mK/√Hz@0.01 Hz and 100 μK/√Hz@0.5 Hz within a temperature measurement range of 15 °C to 40 °C.

### 3.6. In Situ Temperature Measurement of a MEMS High-Precision Accelerometer

**Figure 11:**
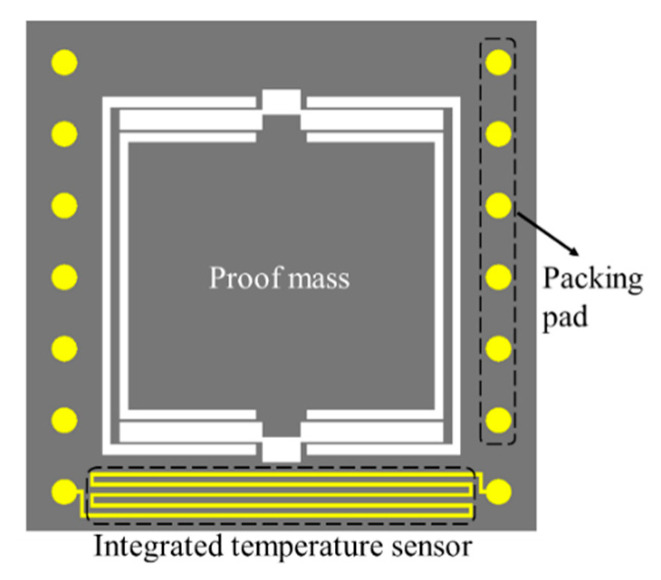
Microelectromechanical systems (MEMS) accelerometer with integrated gold-film temperature sensor.

The proposed gold-film temperature sensor was integrated on a MEMS high-precision accelerometer for in situ temperature measurement of the acceleration sensing element, as shown in Figure 11. The reported MEMS high-precision accelerometer [18] consists of a silicon-based spring-mass system fabricated by through-wafer etching process [19] and matching electrodes on both the mass surface and the upper glass layer for area-change capacitive displacement transduction. The gold-film temperature sensor was fabricated on the accelerometer frame simultaneously with the capacitive electrodes.

**Figure 12:**
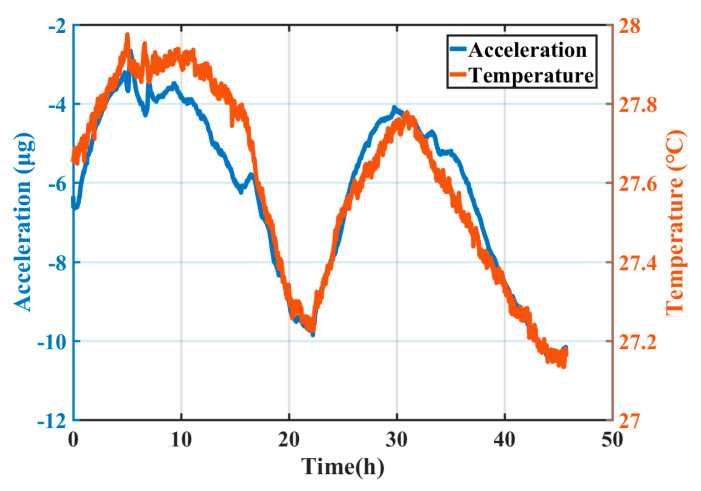
Changes in acceleration output and temperature output over time.

**Figure 13:**
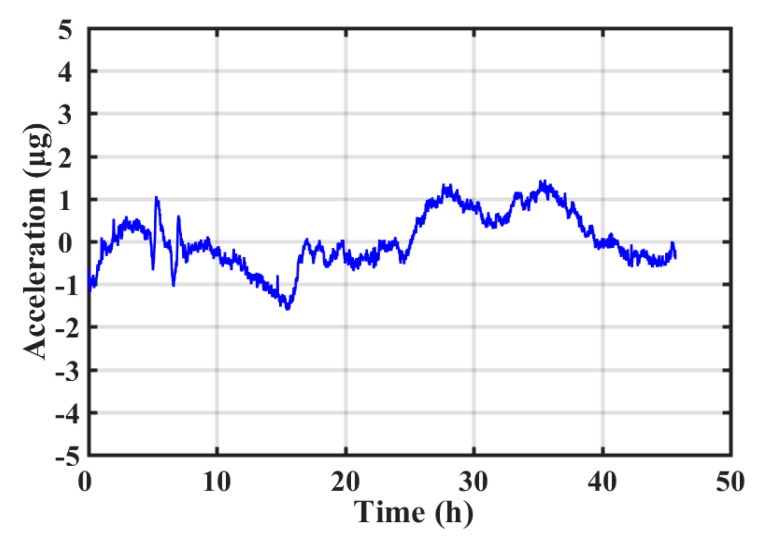
Stability of the MEMS accelerometer with thermal effect deducted and offset removed.

Since the silicon-based MEMS accelerometer had a much larger temperature coefficient of Young’s modulus than the commonly used quartz-based high-precision accelerometers, in order to obtain high-precision performance, the thermal drift of the MEMS accelerometer needed to be minimized. The MEMS high-precision accelerometer with the integrated temperature sensor was placed in a passive thermal insulation box for long-term stability test in the lab with the temperature controlled by the air conditioner. The integrated temperature sensor was for in situ monitoring temperature change of the accelerometer. The stability test lasted for 46 h with a sampling rate of 1 Hz. The output of the MEMS accelerometer is illustrated in Figure 12 and the stability could be calculated to be 2.15 μg (1σ). The temperature change measured by the integrated temperature sensor is also shown in Figure 12. The correlation coefficient between acceleration and temperature was as high as 0.95. Therefore, the first-order linear fitting between the acceleration output and the temperature output was performed. The goodness of fit was 0.91 and the temperature coefficient of the MEMS accelerometer was 8.608 μg/°C. After deducting the temperature effect, the stability of the MEMS accelerometer could be reduced to 640 ng (1σ), as shown in Figure 13. It can be seen that the on-chip integrated gold-film temperature sensor could correlate the acceleration caused by temperature change, further improving the stability of the MEMS high-precision accelerometer.

## 4. Discussion

The proposed temperature sensor can work under either a wide temperature range or a narrow range, depending on the application requirement. For less precise acceleration measurement, the accelerometer is generally not under temperature control; therefore, the temperature effect is suppressed by measuring the large environment temperature fluctuation and then conducting compensation procedure. However, for high-precision acceleration measurement, the accelerometer is generally under passive or active temperature control with the interior temperature fluctuation of a few degrees or within one degree Celsius. The task of the on-chip temperature sensor is to conduct in situ and high-resolution temperature measurement within a small fluctuation, which is the essence of the precise temperature compensation. The proposed temperature sensors are calibrated and demonstrated for this task; however, it can also be used for other applications by tuning the measurement range. Figure 12 shows acceleration and temperature fluctuations at the working temperature point of close to room temperature in the lab. However, in practical application, the temperature sensor should be configured to have optimized performance (sensitivity, linearity, etc.) at the certain system working temperature point by adjusting the setting resistors of the circuit as shown in Figure 4.

For traditional temperature compensation of MEMS sensors, separated thermistors in the chip carrier are generally used, or temperature information from the circuit components, such as the microcontroller, is used with a typical resolution of one degree Celsius or worse. Therefore, the temperature compensation errors come from the temperature gradient, time constant and measurement resolution. However, the proposed integrated gold-film temperature sensor can perform in situ temperature measurement with a resolution of about 0.1 mK and ignorable time lag. In addition, the fabrication process is compatible with most MEMS processes; therefore, the integrated temperature sensor can be applied on most MEMS sensors without much cost and room request.

The resolution of the proposed temperature sensor was about four parts per million of the temperature measurement span of 25 °C. The essence of achieving the performance lies in the dedicated thermistor signal amplifier INA330. First, compared with the classical bridge circuit, the INA330 circuit had less mismatching problem in terms of resistance values and temperature coefficients of resistance. Second, the temperature coefficient of its offset voltage was very small, only 0.2 µV/°C. Third, its 1/f noise at low-frequency range was small. Therefore, the INA330 circuit had excellent performance in terms of noise rejection.

Apart from the temperature effect, the stability of a MEMS accelerometer is also affected by other effects, such as stress. In practices, MEMS accelerometers are usually thermal cycled to release most of the stress before functioning.

## 5. Conclusions

In this study, we demonstrate an integrated resistor-type temperature sensor. The fabricated temperature sensor prototypes were first calibrated and then tested for related performance, including consistency, repeatability, noise floor, etc. Experimental results show that the temperature measurement accuracy was 0.08 °C; the discrepancies among the sensors were within 0.02 °C; the repeatability within seven days was 0.03 °C; the noise floor was 1 mK/√Hz@0.01 Hz and 100 μK/√Hz@0.5 Hz. Since the proposed on-chip temperature sensor use MEMS compatible fabrication processes and low-noise signal conditioning circuit, it was capable of in situ and high-resolution temperature measurement of the main MEMS sensor. It was promising to solve the issues of temperature gradient, thermal transmission time and low measurement resolution that were the main errors of traditional temperature compensation for high-precision MEMS sensors. The integration test with a MEMS accelerometer showed that by employing the temperature sensor, and subtracting the temperature effects, the bias stability within 46 h of the MEMS accelerometer was improved from 2.15 μg to 640 ng with three times improvement on magnitude. Due to its simplicity and compatibility in design and fabrication, the proposed temperature sensor could be easily integrated with other MEMS sensors.

## Figures and Tables

**Table 1:** Design parameters of gold-film temperature sensor.

Chip Type	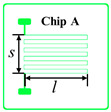	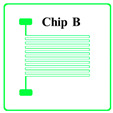	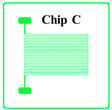	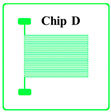	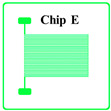
*N*	10	20	30	40	50
*L* (mm)	36.3	69.0	101.7	134.4	167.1
Calculated resistance value (Ω)	871	1656	2440	3225	4010
Measured resistance value (Ω)	900.2	1800.7	2701.1	3601.8	4502.3

**Table 2:** Parameters of the temperature sensors used in different experiments.

No.	Experiment	Chip Type	Quantity
1	Calibration test	Chip C	2
		Chip D	2
		Chip E	3
2	Consistency study	Chip E	3
3	Repeatability study	Chip E	1
4	Measurement resolution	Chip E	1
